# Persistent symbiont colonization leads to a maturation of hemocyte response in the *Euprymna scolopes*/*Vibrio fischeri* symbiosis

**DOI:** 10.1002/mbo3.858

**Published:** 2019-06-13

**Authors:** Bethany Rader, Sarah J. McAnulty, Spencer V. Nyholm

**Affiliations:** ^1^ Department of Microbiology Southern Illinois University Carbondale Illinois; ^2^ Department of Molecular and Cell Biology University of Connecticut Storrs Connecticut

**Keywords:** bacteria, *Euprymna scolopes*, hemocyte, phagocytosis, symbiosis, *Vibrio fischeri*

## Abstract

The binary association between the squid, *Euprymna scolopes*, and its symbiont, *Vibrio fischeri*, serves as a model system to study interactions between beneficial bacteria and the innate immune system. Previous research demonstrated that binding of the squid's immune cells, hemocytes, to *V. fischeri* is altered if the symbiont is removed from the light organ, suggesting that host colonization alters hemocyte recognition of *V. fischeri*. To investigate the influence of symbiosis on immune maturation during development, we characterized hemocyte binding and phagocytosis of *V. fischeri* and nonsymbiotic *Vibrio harveyi* from symbiotic (sym) and aposymbiotic (apo) juveniles, and wild‐caught and laboratory‐raised sym and apo adults. Our results demonstrate that while light organ colonization by *V. fischeri* did not alter juvenile hemocyte response, these cells bound a similar number of *V. fischeri* and *V. harveyi* yet phagocytosed only *V. harveyi*. Our results also indicate that long‐term colonization altered the adult hemocyte response to *V. fischeri* but not *V. harveyi*. All hemocytes from adult squid, regardless of apo or sym state, both bound and phagocytosed a similar number of *V. harveyi* while hemocytes from both wild‐caught and sym‐raised adults bound significantly fewer *V. fischeri*, although more *V. fischeri* were phagocytosed by hemocytes from wild‐caught animals. In contrast, hemocytes from apo‐raised squid bound similar numbers of both *V. fischeri* and *V. harveyi*, although more *V. harveyi* cells were engulfed, suggesting that blood cells from apo‐raised adults behaved similarly to juvenile hosts. Taken together, these data suggest that persistent colonization by the light organ symbiont is required for hemocytes to differentially bind and phagocytose *V. fischeri*. The cellular immune system of *E. scolopes* likely possesses multiple mechanisms at different developmental stages to promote a specific and life‐long interaction with the symbiont.

## Graphical abstract

Using the *Euprymna scolopes*/*Vibrio fischeri* symbiosis, we analyzed hemocyte binding and phagocytosis of *V. fischeri* and the nonsymbiotic bacterium *V. harveyi* in (a) colonized (symbiotic; sym) and uncolonized (aposymbiotic; apo) juvenile animals, (b) wild‐caught naturally sym adult animals, (c) animals raised sym, and (d) apo. We report that while hemocytes from juvenile animals do not alter their response to *V. fischeri* based on symbiont colonization state, they do distinguish between *V. fischeri* and *V. harveyi* while showing a general increase in binding to both species, and that long‐term colonization is required for hemocytes to differentially recognize the symbiont. In addition, we report that initial tolerance of the symbiont appears to occur at the level of phagocytosis, whereas later tolerance is achieved at the level of hemocyte binding.

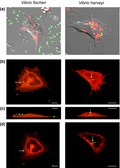

## 1 INTRODUCTION

Most if not all metazoans enter into life‐long beneficial interactions with microorganisms. These symbionts are in constant contact with, and have helped shape the evolution of the immune systems of their hosts (Dishaw & Litman, 2013; Gross et al., 2009; Loker, 2012; McFall‐Ngai, 2007; McFall‐Ngai, Nyholm, & Castillo, 2010). Invertebrates comprise the majority of metazoan life on the planet, and many members from this group form highly specific associations with microbes, ranging from binary relationships with one microbial partner to complex consortia (Cavanaugh, McKiness, Newton, & Stewart, 2013; Collins, LaBarre, et al., 2012; Hussa & Goodrich‐Blair, 2013; Moran, 2006; Nyholm & Graf, 2012). However, unlike vertebrates, invertebrates lack a canonical adaptive immune system and antibody response that is synonymous with specific responses to microbes. Recently, the innate immune system has been implicated in the establishment and maintenance of these associations, and invertebrate model systems are proving valuable for exploring interactions between beneficial microbes and the innate immune system (Bosch, 2014; Chu & Mazmanian, 2013; Collins, Schleicher, Rader, & Nyholm, 2012; Douglas, 2014; Ryu, Ha, & Lee, 2010; You, Lee, & Lee, 2014).

**Figure 1:**
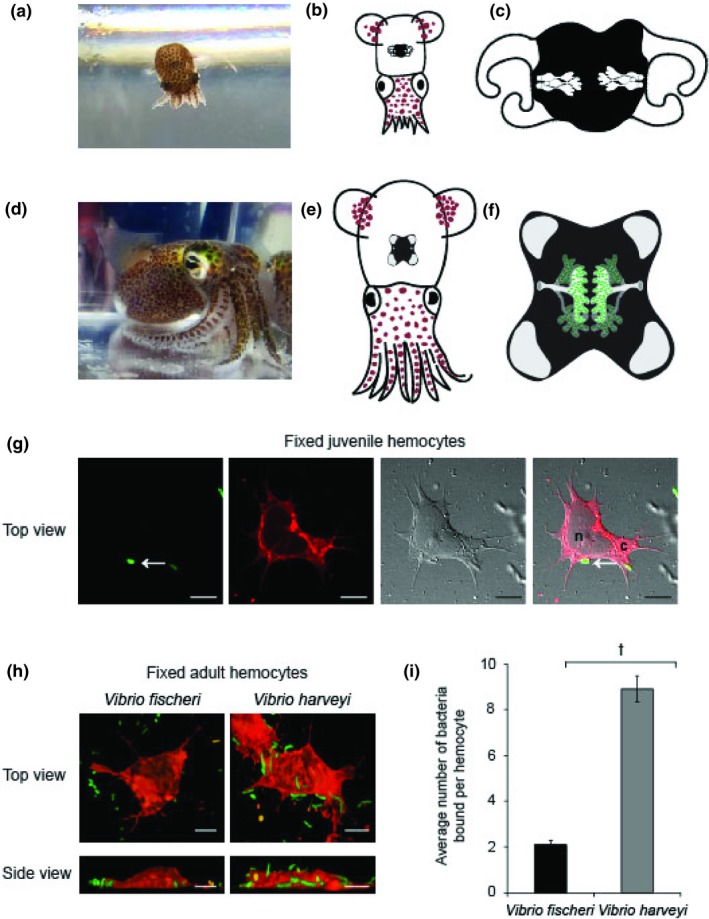
*Euprymna scolopes* produces one type of circulating macrophage‐like blood cell (hemocytes) that differentially bind bacteria. (a,d) Photographs of a juvenile and an adult *E. scolopes* respectively. (b,e) Cartoon depictions of the light organ in reference to the morphology of juvenile (b; hatchling) and adult *E. scolopes* (e). (c,f) Diagrams of the internal architecture of uncolonized hatchling and colonized adult light organs showing crypt spaces that house *Vibrio fischeri*. (g) Confocal micrographs of a hemocyte isolated from juvenile animals, adhered to a glass surface and fixed. A GFP expressing *V. fischeri* cell (arrows, green) was bound to the hemocyte cell membrane stained with Concanavalin A (Con A, red), and observed with differential interference contrast (DIC) microscopy, scale, 10 µm. n, nucleus; c, cytoplasm (h) Confocal micrographs of fixed hemocytes isolated from symbiotic wild‐caught adult animals bound to GFP‐expressing *V. fischeri* or *V. harveyi.* (i) The average number of *V. fischeri* or *V. harveyi* bound per hemocyte. Bars represent the average (±*SE*) of three biological replicates (10 hemocytes per replicate). ^†^Levels of bacterial binding that are significantly different from each other (Students *t* test *p* < 0.001)

The light organ symbiosis between the Hawaiian bobtail squid *Euprymna scolopes* and the bioluminescent Gram‐negative marine bacterium *Vibrio fischeri* is a model system for investigating the mechanisms by which beneficial host–microbe associations are established and maintained (reviewed in McFall‐Ngai, 2008; Nyholm & McFall‐Ngai, 2004; Figure 1a–f). Remarkably, the host recruits only *V. fischeri* from a background of 10^6^ nonsymbiotic bacteria per milliliter of seawater (reviewed in McFall‐Ngai, 2014; Nyholm & McFall‐Ngai, 2004). This specificity is maintained during the life of the host despite the light organ being in direct contact with the environment via ciliated ducts that facilitate venting of the symbionts as part of a daily rhythm (Figure 1c,f). This venting regulates the association and seeds the environment with viable *V. fischeri* for the next generation of squid. There are a number of both host and symbiont factors that promote specificity and the host's innate immune system has been identified as a major contributor to the association (McFall‐Ngai et al., 2010). These factors include the recognition of microbe‐associated molecular patterns (MAMPs) by pattern recognition receptors (PRRs) (Troll et al., 2009, 2010), production of reactive oxygen species (ROS) (Davidson, Koropatnick, Kossmehl, Sycuro, & McFall‐Ngai, 2004; Weis, Small, & McFall‐Ngai, 1996), modification of symbiont bioreactive molecules (Heath‐Heckman et al., 2014; Rader, Kremer, Apicella, Goldman, & McFall‐Ngai, 2012; Troll et al., 2010), involvement of immune pathways such as Toll‐mediated NF‐kB and complement pathways (Castillo, Goodson, & McFall‐Ngai, 2009; Collins, Schleicher, et al., 2012; McFall‐Ngai et al., 2010), and symbiont interactions with the host's blood cells (Nyholm & Mcfall‐Ngai, 1998; Nyholm, Stewart, Ruby, & McFall‐Ngai, 2009).

*Euprymna scolopes* produces a single type of macrophage‐like hemocyte that can migrate between tissues (Heath‐Heckman & McFall‐Ngai, 2011; Koropatnick, Kimbell, & McFall‐Ngai, 2007; Nyholm & Mcfall‐Ngai, 1998), and bind and phagocytose bacteria both in vivo and in vitro (Nyholm & Mcfall‐Ngai, 1998; Nyholm et al., 2009, Figure 1g–i). Hemocytes migrate into the light organ as early as 2 hr post‐hatching upon detection of tracheal cytotoxin (TCT), a monomer of peptidoglycan (Koropatnick et al., 2004, 2007). Hemocytes are detected in the luminal spaces of both juvenile and adult light organs where they are thought to sample the bacterial contents, and release chitin that is metabolized by the symbiont in adult hosts (Heath‐Heckman & McFall‐Ngai, 2011; Koropatnick et al., 2007; Nyholm & Mcfall‐Ngai, 1998; Schwartzman et al., 2015). Several studies support the hypothesis that colonization of the light organ influences the maturation of hemocytes and the ability to recognize *V. fischeri*. Using proteomics and transcriptomics, two studies identified innate immune proteins/genes that were differentially abundant/expressed in hemocytes isolated from wild‐caught sym animals as compared to animals that had been cured of *V. fischeri* using antibiotics (Collins, Schleicher, et al., 2012; Schleicher, VerBerkmoes, Shah, & Nyholm, 2014). A third study found that hemocytes from colonized adult hosts differentially bound various *Vibrio* species, adhering to five times more *V. fischeri* cells when isolated from animals in which the light organ had been cured of the symbiont (Nyholm et al., 2009). Taken together, these studies suggest that colonization of the light organ initiates a maturation program that results in the hemocytes’ ability to recognize and differentially respond to the symbiont. Because these previous studies were all conducted using hemocytes from adult animals, it remains unclear to what extent any maturation process begins at hatching, when *V. fischeri* are recruited from the seawater and specificity is first initiated.

In this study, we sought to expand our understanding of hemocyte maturation and specificity by examining binding and phagocytosis behavior of hemocytes from symbiotic (sym; colonized) and aposymbiotic (apo; uncolonized) juvenile animals over the first 96 hr of the association. We also investigated whether long‐term light organ colonization affects hemocyte maturation in apo and sym animals raised to sexual maturity. We report that while juvenile hemocytes do not alter their response to the symbiont based on colonization state, they do distinguish between two closely related *Vibrio* species: *V. fischeri* and *Vibrio harveyi*. We also report that long‐term colonization is required for hemocytes to differentially recognize the symbiont. These data suggest that juvenile squid hatch with an immune system primed to initiate colonization with an attenuated immune response toward the symbiont, perhaps preserving a “naïve” state in which *V. fischeri* can colonize without immune repercussions.

## 2 METHODS

### 2.1 Animal culture

Adult *E. scolopes* were collected in shallow water off the shores of Oahu Hawaii and bred and maintained as previously described (Montgomery & McFall‐Ngai, 1993) in an aquatic facility containing recirculating artificial seawater at the University of Connecticut (Schleicher & Nyholm, 2011). Juvenile animals were colonized with *V. fischeri* ES114 by addition of 3,000 colony forming units per milliliter (CFUs/ml) to filter‐sterilized artificial seawater (FSASW) for up to 24 hr. Colonization by *V. fischeri* was determined by monitoring luminescence using a FB12 single tube luminometer (Titertek Berthold, Huntsville Alabama).

### 2.2 Aposymbiotic juveniles raised to adulthood

Newly hatched juvenile squid were raised in FSASW as described in (Koch, Miyashiro, McFall‐Ngai, & Ruby, 2014). After approximately 6–8 weeks and once feeding exclusively on glass shrimp, the juveniles were moved to tanks with recirculating ASW and were exposed to the same laboratory conditions as wild‐caught animals. The animals were maintained in these tanks for 4–6 more weeks prior to sacrifice, at which time the animals had reached adult size having mantle lengths between 21 and 25 mm, similar to wild‐caught adults. Dissections post‐bleeding confirmed that these animals were sexually mature. To confirm that the light organs of the apo‐raised adults were not colonized by *V. fischeri*, at the time of sacrifice one lobe of the light organ central core was removed, homogenized in sterile marine phosphate‐buffered saline (mPBS—50 mM sodium phosphate buffer with 0.45 M NaCl, pH 7.4), serially diluted in mPBS and plated on LBS (Luria–Bertani salt medium) agar. Plates were incubated overnight at 28**°**C confirming the absence of countable CFUs.

### 2.3 Isolation of host hemocytes

Isolation of adult hemocytes was performed as previously reported (Nyholm et al., 2009). Adult animals were anesthetized in 2% ethanol in FSASW and hemolymph was drawn from the cephalic blood vessel located between the eyes using a 1‐ml syringe with a 27‐gauge needle, providing approximately 3,000–5,000 hemocytes/µl. Hemocytes were washed three times and resuspended in Squid‐Ringer's solution (SR; 530 mM NaCl, 10 mM KCl, 25 mM MgCl_2_, 10 mM CaCl_2_, and 10 mM HEPES buffer, pH 7.5). Hemocytes were spread evenly among wells in either an 8‐well chambered borosilicate cover glass (Thermo Fisher Scientific, Waltham, MA) for live imaging, or a 12‐well plate to which circular No. 1 glass coverslips had been added to the wells for fixed imaging. Isolation of juvenile hemocytes was performed as previously reported (Heath‐Heckman & McFall‐Ngai, 2011). Juvenile animals were anesthetized in 2% ethanol in FSASW. The FSASW was replaced with SR and the animals were homogenized. The liquid homogenate was then added to either an 8‐ or 12‐well chambered borosilicate cover glass and were allowed to adhere for 30 min. Each well contained the homogenate from 10 juvenile squid. Hemocytes were washed three times for 10 min in SR. Juvenile hemocyte extractions yielded approximately 100–150 hemocytes per animal.

### 2.4 Preparation of bacterial cultures and bacterial binding assays

Bacteria were grown in LBS, with 20 µg/ml chloramphenicol for GFP strains, for 3–4 hr at which point the OD600 was measured and cells were pelleted and resuspended in SR to a concentration of 1 ×10^8^ ml^−1^. Strains used in this study are listed in Table 1. Bacteria were added to hemocytes adhered to coverslips at a ratio of 50 bacteria per hemocyte. The hemocytes plus bacteria were incubated for 1 hr, at which time they were washed three times in SR to remove unbound bacteria. To determine the number of bacteria bound, 4% paraformaldehyde in SR was added to each cover slip in the 12‐well dish and incubated shaking for 30 min. The coverslips were then washed in 1× PBS four times for 5 min each and stored at 4**°**C until use.

**Table 1:** Bacterial strains used in this study

Strain	Plasmid	Reference
*Vibrio fischeri* Es114		Boettcher and Ruby (1990)
*V. fischeri* Es114	pKV111	Nyholm, Stabb, Ruby, and McFall‐Ngai (2000)
*V. harveyi* B392	pKV111	Nyholm et al. (2009)

### 2.5 Visualization of hemocytes and bacteria

Fixed hemocytes were incubated in permeabilization buffer (1× PBS containing 1% Triton‐X) for 30 min then counterstained overnight with 1 µg/ml Concanavalin A (ConA; Life Technologies, Grand Island, NY) in permeabilization buffer. Cover slips were washed four times for 5 min each in 1× PBS and mounted onto a glass slide with Vectashield (Vector Laboratories, Burlingame, CA) and sealed with nail polish. For live imaging of bacterial cell internalization, pHrodo Bioparticles (Life Technologies) which fluoresce in phagosomes were prepared by sonication and added to the washed chamber wells at a concentration of 10 µg/ml. Cells fluorescently labeled either with ConA or pHrodo (red) and green fluorescent protein (GFP)‐producing bacteria (green) were visualized using a Nikon A1R laser‐scanning confocal microscope (Nikon Corp., Tokyo, Japan) at the Flow Cytometry and Confocal Microscopy Facility at the University of Connecticut. Images were analyzed using FIJI (http://fiji.sc/).

### 2.6 Statistical analysis

**Figure 2:**
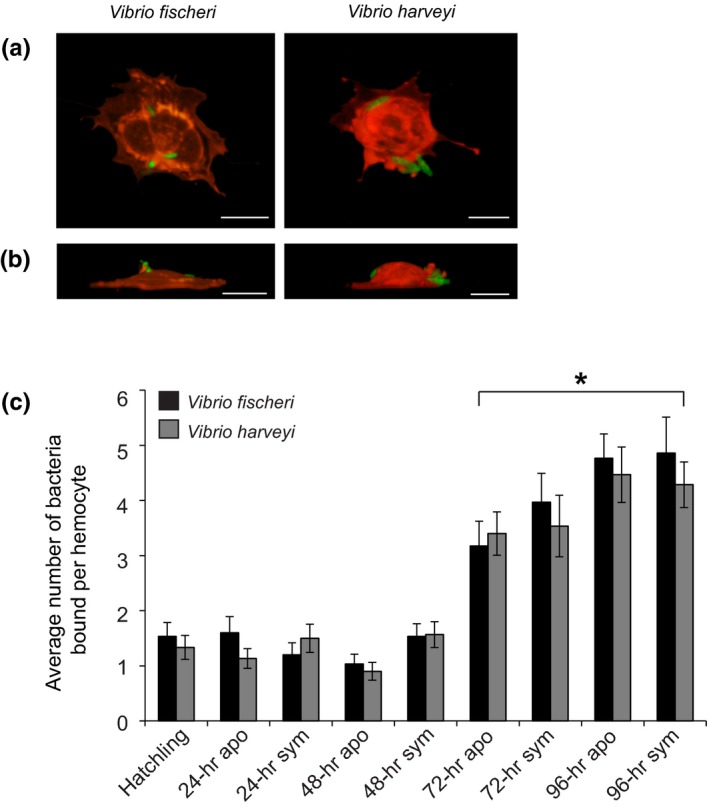
Binding of *Vibrio fischeri and Vibrio harveyi* to juvenile hemocytes. (a, b) Representative 3D confocal images of bacteria bound to juvenile hemocytes were constructed from confocal Z‐series showing GFP‐expressing *V. fischeri or V. harveyi* (green) and hemocyte counterstaining with Con A (red), scale, 10 µm. (a) Top view of 3‐D images. (b) Side view of 3D images, with the adherent side of the hemocytes at the bottom of the images. (c) The average number of bacteria bound per hemocyte. Bars represent the average (±*SE*) of three biological repeats of 10 hemocytes chosen randomly from 10 different microscopic fields from a preparation of isolated hemocytes from 10 pooled animals (*n* = 4). *Time points that were statistically significantly different than the 0‐hr time point (ordinary least squares regression, *p* < 0.001)

Data analyzed were from independent samples comprised of hemocytes isolated from individual adult animals, or groups of 10 juvenile animals randomly selected from hatching egg clutches. A Student's *t* test was performed to analyze the difference between hemocyte binding of *V. fischeri* and *V. harveyi* during fixation protocol development (Figure 1i). To analyze the juvenile hemocyte data, we conducted an ordinary least squares regression using the R statistical computing platform version 3.5.1 with the tidyverse library in the RStudio integrated development environment, with the number of bound bacteria as the dependent variable, and the time interval since hatching, the type of bacteria, and whether the squid were apo or sym as the predictors in the model (Figure 2c). The intercept in the model is the category for 0 hr (hatchling), *V. fischeri*, and apo squid, and the results of the analysis are shown in Table 2. We conducted a two‐factor ANOVA using GraphPad PRISM on all subsequent data to determine if there is an interaction between the independent variables (squid symbiotic state [Figure 4a,b apo‐raised, sym‐raised, and sym‐wild] and bacterial species [*V. harveyi* or *V. fischeri*, Figure 4a,b]) and the dependent variables (bacterial binding [Figure 4a] or bacterial phagocytosis [Figure 4b]). Post hoc pairwise comparisons using the Sidak's correction for multiple comparisons were used to compare means within the independent variables for Figure 4a,b.

**Table 2:** Regression analysis results

Category	Dependent variable: number of bacteria bound
Time interval: 24 hr versus 0 hr	−0.209 (0.314)
Time interval: 48 hr versus 0 hr	−0.309 (0.314)
Time interval: 72 hr versus 0 hr	1.966 (0.314)^a^
Time interval: 96 hr versus 0 hr	3.084 (0.324)^a^
Bacteria: *Vibrio harveyi* versus *V. fischeri*	−0.154 (0.169)
sym state: apo versus sym	0.267 (0.182)
Constant	1.510 (0.260)^a^
Observations	508
*R* ^2^	0.340
Adjusted *R* ^2^	0.332
Residual *SE*	1.903 (*df* = 501)
*F* Statistic	43.014 (*df* = 6; 501)^a^

Abbreviations: apo, aposymbiotic; sym, symbiotic.

a *p* < 0.001.

## 3 RESULTS

### 3.1 Binding of *Vibrio* bacterial cells to juvenile hemocytes

To understand whether *E. scolopes* hemocytes are conditioned to distinguish *V. fischeri* from other *Vibrio* species during the first 96 hr of colonization, we first sought to modify the live hemocyte methodology described in (Nyholm et al., 2009), wherein hemocytes isolated from adult animals were visualized live within an hour and a half of isolation. The small number of hemocytes extracted per juvenile animal (~100) and the large number of animals/blood samples collected over multiple days and over multiple animal cohorts that were required for hemocyte analyses made it unfeasible to do live imaging directly after hemocyte extraction. Therefore, we fixed juvenile hemocytes that had been exposed to bacteria for later visualization via confocal microscopy (Methods; Figure 1g,h). We tested this fixation method first on hemocytes isolated from wild‐caught adult animals as a proof‐of‐principle, repeating the experiment previously published (Nyholm et al., 2009, Figure 1i). We focused on *V. fischeri* and *V. harveyi* since these species had previously been shown to have the largest difference in hemocyte binding (Nyholm et al., 2009). Using fixed hemocytes resulted in the same trend as was reported for live hemocyte binding assays (Nyholm et al., 2009) whereby significantly more *V. harveyi* were bound by hemocytes compared to *V. fischeri* (Figure 1i).

To identify any differences in bacterial binding during the first 96 hr of light organ colonization, we isolated hemocytes from animals at 0 hr (hatching), and apo and sym animals at 24, 48, 72, and 96 hr post‐hatching, and quantified the number of bound *V. fischeri* and *V. harveyi* cells (Figure 2a,b). Regression analysis showed the number of bacteria bound to hemocytes did not differ depending on which type of bacteria the hemocytes interacted with, or whether the hemocytes were extracted from apo or sym hatchlings. However, there was a statistically significant difference in the number of bound bacteria between the 0‐hr time point compared to the 72‐hr and 96‐hr time points (Figure 2c), suggesting an increased general hemocyte response to bacteria over early development.

### 3.2 Juvenile hemocytes differentially phagocytose bacteria

**Figure 3:**
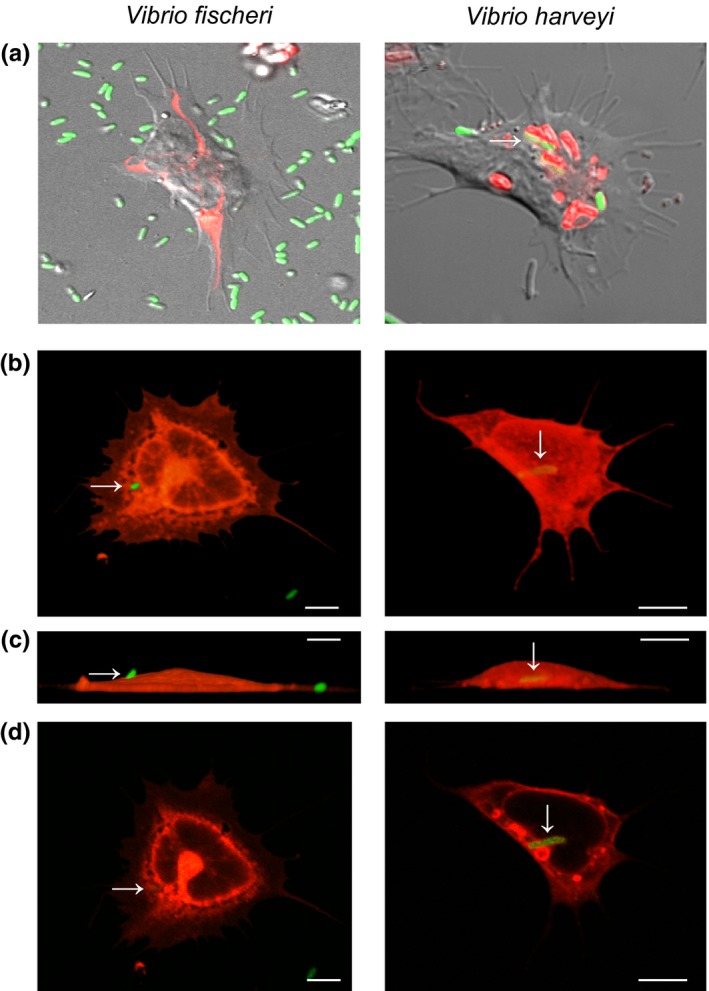
Phagocytosis of *Vibrio fischeri and V. harveyi* by juvenile hemocytes. (a) DIC confocal images of live juvenile hemocytes bound to and/or internalizing GFP‐expressing bacteria (green), and colocalized with acidic compartments (red, pHrodo pH indicator). (b–d) Confocal images of fixed juvenile hemocytes (red, Con A) bound to and/or internalizing GFP‐expressing bacteria (arrows, green), scale, 10 µm. (b) Top view, 3D images. (c) Side view, 3D images, with the adherent side of the hemocyte at the bottom of the images. (d) Image section through the middle of the cell, to confirm internalization of bacteria

To determine if juvenile hemocytes phagocytose *V. fischeri* and *V. harveyi*, we first conducted live imaging of blood cells isolated from 24‐hr sym juvenile animals that were incubated with either *V. fischeri* or *V. harveyi* plus a dye conjugate that is phagocytosed with the bacteria and fluoresces in acidic cellular compartments to confirm internalization of bacteria into hemocyte phagosomes. We observed colocalization of GFP (bacteria) and dye conjugate (putative phagosomes) for *V. harveyi* but not *V. fischeri* (Figure 3a). After fixing cells, bacteria were localized within hemocytes by creating three‐dimensional renderings of hemocytes and localizing GFP‐labeled bacteria within the cell membranes (Figure 3b–d, Table 3). We observed the presence of *V. harveyi* in juvenile hemocytes regardless of symbiotic state or the time point at which they were isolated. Overall, an average of 30% of the hemocytes observed contained phagocytosed *V. harveyi. Vibrio fischeri* remained external and was not phagocytosed, indicating that upon hatching, the hemocytes can distinguish between these two different *Vibrio* species, and/or *V. fischeri* has mechanisms to evade phagocytosis at this developmental stage.

**Table 3:** Summary of internalization of *Vibrio harveyi* by juvenile hemocytes

Time‐point	Total number of hemocytes per treatment per bacterial exposure^a^	Number of hemocytes containing phagocytosed *Vibrio fischeri*	Number of hemocytes containing phagocytosed *V. harveyi*	Percentage of hemocytes containing phagocytosed *V. harveyi*	Number of *V. harveyi* visualized phagocytosed per hemocyte ± *SE*
Hatchling	30	0	8	26.6	1.25 ± 0.46
24‐hr apo	30	0	13	43.3	1.62 ± 0.96
24‐hr sym	30	0	7	13.3	1.29 ± 0.49
48‐hr apo	30	0	10	33.3	1.80 ± 1.03
48‐hr sym	30	0	9	30.0	1.77 ± 1.09
72‐hr apo	30	0	10	33.3	1.77 ± 1.09
72‐hr sym	30	0	8	26.6	1.25 ± 0.47
96‐hr apo	30	0	11	36.3	1.45 ± 0.69
96‐hr sym	30	0	9	30.0	1.44 ± 0.53

Abbreviations: apo, aposymbiotic; sym, symbiotic.

a Phagocytosed bacteria were counted from hemocytes visualized in Figures 2 and 3.

### 3.3 Hemocytes from adults raised without *V. fischeri* differ in their response to bacterial challenge compared to hemocytes from sym‐raised and wild‐caught sym adults

**Figure 4:**
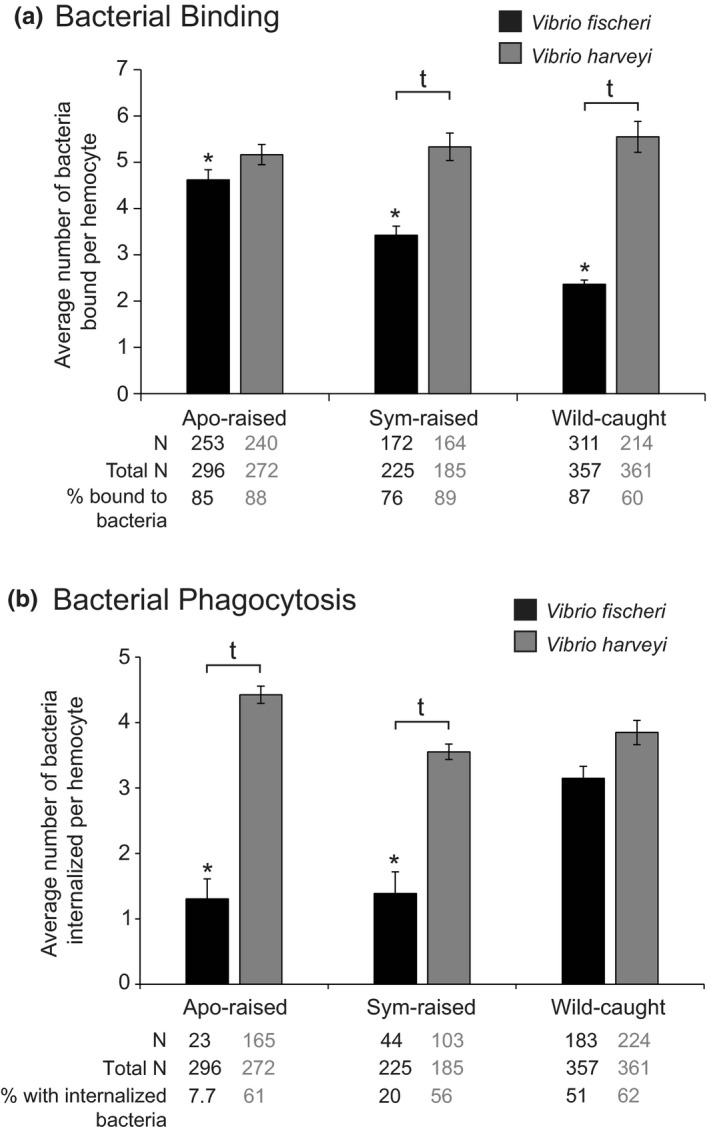
Binding and phagocytosis of *Vibrio fischeri* and *Vibrio harveyi* to hemocytes isolated from aposymbiotic(apo)‐raised, symbiotic (sym)‐raised, and wild‐caught adult animals. (a) The average number of bacteria bound per hemocyte. ^†^Levels of bacterial binding that were significantly different from each other within an animal treatment, *p* < 0.0001. *Levels of *V. fischeri* binding that were significantly different from all other animal treatments, *p* < 0.001. Statistical significance was determined by two‐way ANOVA with post hoc pairwise comparisons using the Sidak's correction for multiple comparisons. (b) The average number of bacteria phagocytosed per hemocyte. ^†^Levels of bacterial internalization that were significantly different from each other within an animal treatment, *p* < 0.001. *Levels of *V. fischeri* internalization that were significantly different from wild‐caught sym, *p* < 0.05. Statistical significance was determined by two‐way ANOVA with post hoc pairwise comparisons using the Sidak's correction for multiple comparisons. (a, b) Bars represent the average ± *SE*. *N* is number of hemocytes for which binding or phagocytosis was observed, Total *N* is the total number of hemocytes used for analysis representing 5–7 biological replicates with a minimum of 30 hemocytes chosen at random per replicate for analysis

**Figure A1:**
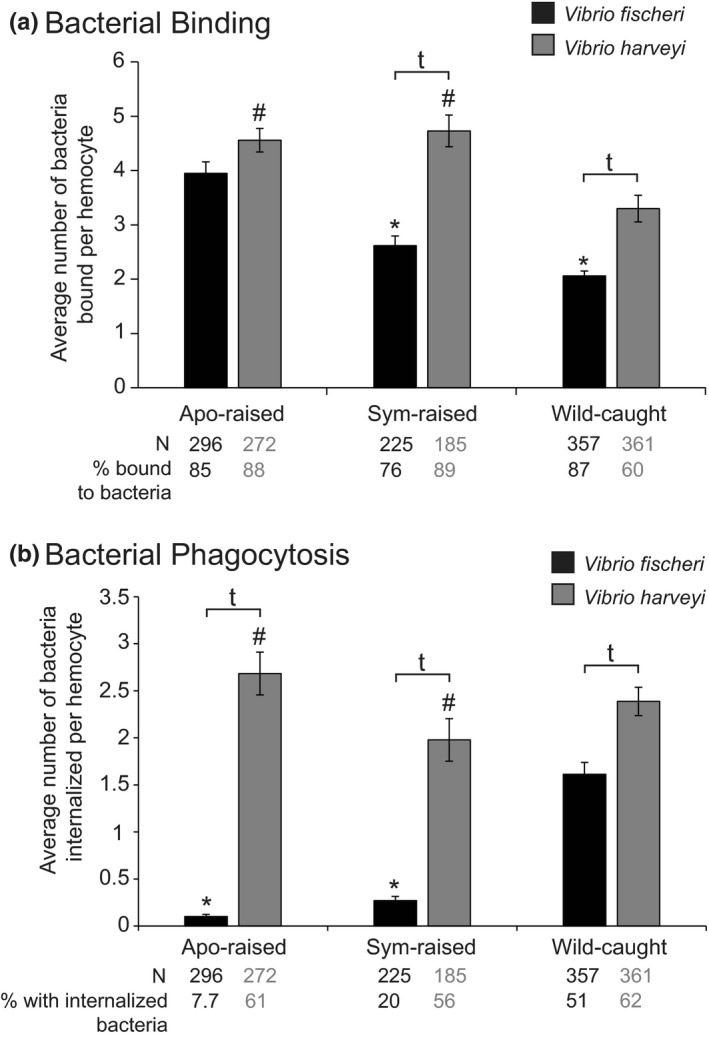
Binding and phagocytosis of *Vibrio fischeri* and *Vibrio harveyi* to hemocytes isolated from aposymbiotic (apo)‐raised, symbiotic‐raised, and wild‐caught adult animals; general hemocyte behavior. (a) The average number of bacteria bound per hemocyte. ^†^Levels of bacterial binding that were significantly different from each other within an animal treatment, *p* < 0.0001. *Levels of *V. fischeri* binding that were significantly different apo‐raised animals but not each other *p* < 0.001. ^#^Levels of *V. harveyi* binding that are significantly different from wild‐caught animals but not each other, *p* < 0.0001. Statistical significance was determined by two‐way ANOVA with post hoc pairwise comparisons using the Sidak's correction for multiple comparisons. (b) The average number of bacteria phagocytosed per hemocyte. ^†^Levels of bacterial internalization that were significantly different from each other within an animal treatment, *p* < 0.0001. *Levels of *V. fischeri* internalization that were significantly different from wild‐caught animals but not each other, *p* < 0.0001. ^#^Levels of *V. harveyi* binding that were significantly different from each other *p* < 0.01. Statistical significance was determined by two‐way ANOVA with post hoc pairwise comparisons using the Sidak's correction for multiple comparisons. (a, b) Bars represent the average ± *SE*. *N* is the total number of hemocytes used for analysis representing 5–7 biological replicates with a minimum of 30 hemocytes chosen at random per replicate for analysis

To understand whether the difference between juvenile and adult hemocyte response to bacterial challenge arises from persistent light organ colonization during postembryonic development, we raised juvenile animals either apo (apo‐raised) or sym (sym‐raised) to sexual maturity. We compared the ability of hemocytes isolated from these animals to bind and phagocytose *V. fischeri* and *V. harveyi* to wild‐caught adults, all of which were sym. Hemocytes were heterogeneous in their interactions with *V. fischeri* and *V. harveyi* with some hemocytes neither binding nor phagocytosing bacteria at a given fixed time point (Appendix Figure A1). This heterogeneity may be because the fixation protocol allowed us to look at binding and phagocytosis behavior at only a single time point after addition of bacteria. In order to discern discriminating hemocyte behavior from general hemocyte behavior, we quantified binding and phagocytosis for those cells that were in direct contact with or contained phagocytosed bacteria (Figure 4). In contrast to sym‐raised and wild‐caught animals, hemocytes from apo‐raised animals bound *V. fischeri* and *V. harveyi* similarly (Figure 4a). Hemocytes from sym‐raised and wild‐caught animals bound significantly fewer *V. fischeri* than apo‐raised, with hemocytes from wild‐caught animals binding the fewest *V. fischeri* cells (Figure 4a). We observed no significant difference in the number of *V. harveyi* bound to hemocytes isolated from apo‐raised, sym‐raised, or wild‐caught animals (Figure 4a). In contrast to sym‐raised and apo‐raised, hemocytes from wild‐caught animals phagocytosed *V. fischeri* and *V. harveyi* similarly (Figure 4b), although we observed a trend of more internalized *V. harveyi* than *V. fischeri*. Hemocytes isolated from apo‐raised animals phagocytosed significantly fewer *V. fischeri* than those from wild‐caught animals, but not sym‐raised animals (Figure 4b). Although the number of phagocytosed *V. fischeri* per hemocyte was similar between apo‐raised and sym‐raised animals, the number of hemocytes with internalized *V. fischeri* in sym‐raised animals was intermediate (20%) compared to apo‐raised (7.7%) and wild‐caught (51%). Similar to bacterial binding, we found no significant difference in the number of *V. harveyi* phagocytosed by hemocytes isolated from apo‐raised, sym‐raised or wild‐caught animals (Figure 4b). Taken together, these binding and internalization data suggest that apo‐raised animals remain in a juvenile‐like state as they bind the same number of *V. fischeri* and *V. harveyi* and phagocytose fewer *V. fischeri* compared to *V. harveyi.* Furthermore, hemocytes from sym‐raised animals may represent an intermediate maturation state; trending toward wild‐caught hosts in binding and phagocytosis behavior.

## 4 DISCUSSION

**Figure 5:**
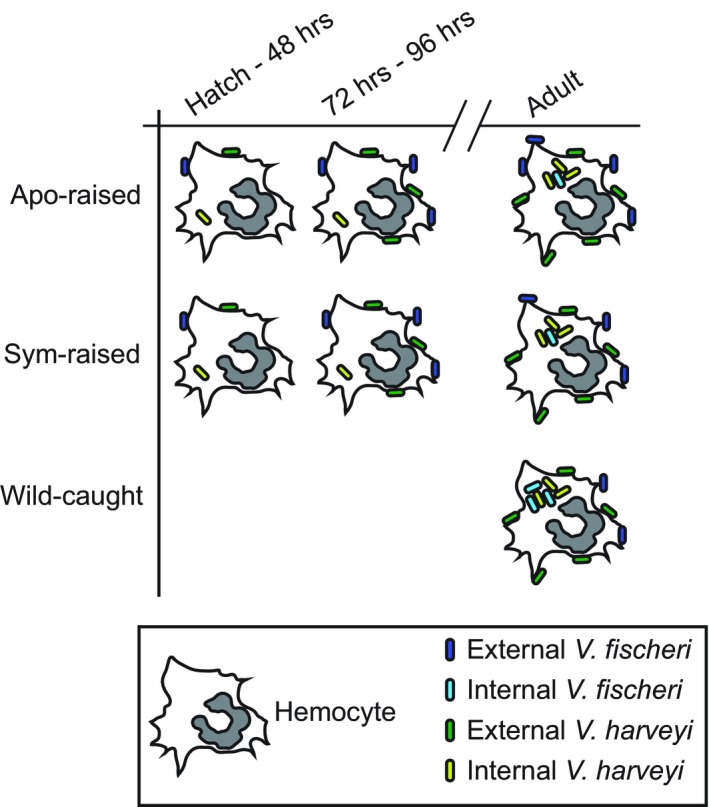
Summary of hemocyte binding and phagocytosis results observed in animals ranging from hatching through adults. The numbers of *Vibrio fischeri* and *V. harveyi* shown attached to (dark blue and dark green, respectively) or inside (light blue and light green respectively) hemocytes correspond to the graphs shown in Figure 2 for juvenile hemocytes and Figure 4 for adult hemocytes

In this study, we report (a) a new method for quantifying hemocyte bacterial binding and phagocytosis in the squid–vibrio system in both juveniles and adults (Figure 1), (b) when challenged with two different *Vibrio* species, hemocytes isolated from juvenile animals bound to symbiotic and nonsymbiotic bacteria similarly during the first 96 hr post‐hatching (Figure 2), (c) at 72 hr post‐hatching, there was an increase in the number of bacteria bound by hemocytes regardless of the symbiotic state of the animal or *Vibrio* species tested (Figure 2), (d) hemocytes isolated from juvenile animals did not phagocytose *V. fischeri* while they did phagocytose *V. harveyi* (Figure 3), (e) hemocytes isolated from adult apo‐raised animals bound a similar number of *V. fischeri* and *V. harveyi* in contrast to sym wild‐caught animals which bound less *V. fischeri* than *V. harveyi* (Figures 1 and 4), (f) hemocytes isolated from apo‐raised adults phagocytosed significantly fewer *V. fischeri* than *V. harveyi* in contrast to wild‐caught sym adults which phagocytosed a similar number of *V. fischeri* and *V. harveyi* (Figure 4), and (g) hemocytes from sym‐raised adult animals showed an intermediate binding and phagocytosis behavior compared with hemocytes from apo‐raised and wild‐caught sym adults (Figure 4). These results are summarized in Figure 5.

### 4.1 Symbiont‐independent hemocyte maturation

The increase in hemocyte binding to bacteria at 72 hr post‐hatching (Figure 2c) suggests that the host's hemocytes undergo systemic maturation that is independent of light organ colonization. This general increase in immune response may result from the differentiation of hemocytes during hematopoiesis, perhaps in response to environmental or microbial cue(s). Hemocyte development and maturation in cephalopods takes place in a specialized hematopoietic organ, the white body, and mature hemocytes are released into the hemolymph (Cowden, 1972 and Claes, 1996). In *Sepia officinalis*, a comprehensive analysis of hemocyte proliferation and maturation showed that the physiology of the white body undergoes a functional shift from proliferation to differentiation as the animal matures (Claes, 1996). Tissues of late embryos and juveniles were mitotically more active and contained a significantly higher percentage of the early blood cell stages. In *E. scolopes*, the 72‐hr time point may also signify a turnover of mature circulating hemocytes, and is consistent with a previous study showing that *V. fischeri* binding by hemocytes isolated from adult animals increased after 3 days of curing (Nyholm et al., 2009). A gene expression study of the white body from *Euprymna tasmanica* also revealed a number of potential genes involved with hematopoiesis (Salazar, Joffe, Dinguirard, Houde, & Castillo, 2015). Future studies will focus on whether hematopoiesis in the *E. scolopes* white body is influenced by bacteria or other environmental cues.

A general maturation of innate immune response toward *V. fischeri* and *V. harveyi* in *E. scolopes* may not be surprising given that immune systems undergoes developmental changes in other organisms. In humans, this includes a general decrease in the number of circulating innate immune cells and a general impairment of functions of these cells such as chemotaxis, phagocytosis, adherence, and migration of those cells in neonates as compared to adults (Georgountzou & Papadopoulos, 2017). Underlying these changes in function are differences in the expression of innate immune genes such as PRRs, lectins, cytokines, and complement, in neonates as compared to adults (Georgountzou & Papadopoulos, 2017; Kollmann, Levy, Montgomery, & Goriely, 2012; Yu et al., 2018). Less is known about general postembryonic innate immune maturation processes in invertebrates. In the ascidian larvae *Boltenia villosa,* specific immune genes showed temporal and spatial patterns of transcription that were restricted to postembryonic morphogenic stages, and blood cell migration occurred in two waves corresponding to changes in larval lifestyle, swimming, and settlement (Davidson & Swalla, 2002). Juvenile animals in our study were not axenic as they were hatched into an environment that, although lacking *V. fischeri* and *V. harveyi*, was not sterile. Therefore, host interactions with other environmental bacteria may also influence innate immune maturation in *E. scolopes*. One strength of the squid–vibrio association is the ability to characterize changes to immune response that are symbiosis dependent or independent without having to maintain animals under germfree conditions.

### 4.2 Symbiont‐dependent hemocyte maturation

Microbe‐associated molecular patterns have been shown to influence cellular development in a number of host–microbe associations, including the squid–vibrio system. For example, exposure to lipopolysaccharide (LPS) led to an increase of proliferating hemocytes in hematopoietic tissue of the black tiger shrimp (*Penaeus monodon*) (van de Braak et al., 2002), and an increase in hemocyte proliferation along with the expression of superoxide dismutase in the freshwater crayfish *Pacifastacus leniusculus* (Wu, Söderhäll, Kim, Liu, & Söderhäll, 2008). In *E. scolopes*, the MAMPs LPS and peptidoglycan derivative, TCT, are responsible for inducing light organ morphogenesis early in postembryonic development (Foster, Apicella, & McFall‐Ngai, 2000; Koropatnick et al., 2004). Tracheal cytotoxin is also responsible for initiating hemocyte trafficking during light organ morphogenesis (Koropatnick et al., 2004, 2007). It is unclear whether MAMPs from *V. fischeri* affect hematopoiesis in the squid. However, *V. fischeri* does influence morphogenic, transcriptional, and proteomic changes in associated light organ tissues and in hemocytes after colonization (Collins, LaBarre, et al., 2012; Kremer et al., 2013; Schleicher et al., 2014; Wier et al., 2010).

Although the mechanisms involved with hemocyte binding and recognition of *V. fischeri* are unknown, they likely involve interactions between host PRRs and their cognate MAMPs. Blood cells from other invertebrates express PRRs for pathogen recognition and ROS and reactive nitrogen species for cytotoxicity (Schmitt et al., 2012). Likewise, hemocytes from *E. scolopes* express PRRs such as *Es*Toll and *Es*PGRP5, as well as members of he conserved NF‐κB signaling pathway, nitric oxide synthase, putative members of the complement pathway, and the carbohydrate‐binding proteins galectins (Collins, Schleicher, et al., 2012; Schleicher et al., 2014). In addition, the transcript and protein abundance of many of these immune factors, as well as protein abundance of genes involved in cytoskeletal dynamics, cell adhesion, and lysosomal function changes depending on symbiotic state of the animal (Collins, Schleicher, et al., 2012; Schleicher et al., 2014). Therefore, it is likely that these changes in gene expression and protein production underlie the molecular differences in binding and phagocytosis reported in this and previous studies (McAnulty & Nyholm, 2017). Our results suggest that hemocyte binding and phagocytosis behavior to *V. fischeri* changes during development (summarized in Figure 5) and future studies should focus on characterizing PRRs during this maturation process.

In addition to MAMPs, *V. fischeri* may have mechanisms to avoid hemocyte binding. *V. fischeri* has an outer membrane protein U (OmpU) that has been implicated in binding to squid hemocytes (Nyholm et al., 2009). OMPs have also been shown to be important virulence factors (McClean, 2012), and mediators of hemocyte–bacteria interactions including *V. harveyi* (Yu, Hu, Sun, & Sun, 2013) and *Vibrio splendidus* infections in oysters (Duperthuy et al., 2011). In *Vibrio parahaemolyticus*, OmpU is regulated by the toxRS two‐component system and is required for stress tolerance and for intestinal colonization of a murine model of infection (Whitaker, Parent, Boyd, Richards, & Boyd, 2012). A previous study also showed that biofilm production by *V. fischeri* may be important in evading hemocyte binding (Pankey et al., 2017). The mechanisms by which OmpU or other symbiont factors contribute to host hemocyte recognition of *V. fischeri* are unknown, but future studies will explore these interactions during host development.

### 4.3 Hemocyte tolerance of the symbiont requires persistent symbiosis

Data from this study suggest that long‐term colonization by *V. fischeri* alters hemocyte response specifically toward the symbiont during post‐embryonic development (Figure 5). Immune cell maturation and development can be influenced by a host's microbiota in a number of ways, often to promote tolerance to symbionts. For example, in vertebrates, bacterial colonization modulates the development of both proinflammatory and anti‐inflammatory T cells in mice (Atarashi et al., 2011; Olszak et al., 2012; Round et al., 2011; Tanoue, Atarashi, & Honda, 2016) and the number of intestinal neutrophils in zebrafish and three‐spined stickleback (Bates, Akerlund, Mittge, & Guillemin, 2007; Kanther et al., 2014; Milligan‐Myhre et al., 2016). In invertebrates, bacterial colonization leads to an increase in the number, circulation, and phagocytic activity of hemocytes in the tsetse fly (Weiss, Wang, & Aksoy, 2011), and decreases the total number of hemocytes and proportion of granulocytes (a hemocyte morphotype) in the pea aphid (Schmitz et al., 2012). In support of the supposition that specific hemocyte response requires persistent symbiont colonization, we found that hemocytes from adult apo‐raised animals bound a similar number of *V. fischeri* and *V. harveyi,* but phagocytosed few *V. fischeri* cells (Figure 4a,b). This finding is in contrast to wild‐caught sym adults that bound fewer *V. fischeri* than *V. harveyi* but phagocytosed a larger number of *V. fischeri* cells when bound (Nyholm et al., 2009, this study). In addition, hemocytes from either apo‐raised or wild‐caught sym adults both bound and phagocytosed a similar number of *V. harveyi* (Figure 4). These data suggest that the change in hemocyte response during maturation was therefore specific toward *V. fischeri*.

In addition to sampling wild‐caught adult hosts, we raised sym squid with the working hypothesis that hemocytes from sym‐raised animals would mirror the wild‐caught animals in hemocyte binding and phagocytic behavior. Hemocytes from sym‐raised animals did bind and phagocytose *V. harveyi* to a similar level as hemocytes from apo‐raised and wild‐caught sym animals (Figure 4a,b). However, they differed in their response to *V. fischeri* from hemocytes from wild‐caught animals in two ways. First, hemocytes from sym‐raised animals bound an intermediate number of *V. fischeri*, statistically fewer than hemocytes from apo‐raised animals and statistically more than hemocytes from wild‐caught animals (Figure 4a). Second, hemocytes from sym‐raised animals phagocytosed a similar number of *V. fischeri* as hemocytes from apo‐raised animals, but fewer than hemocytes from wild‐caught animals (Figure 4b). However, an intermediate number of hemocytes from sym‐raised squid had internalized *V. fischeri* compared to apo‐raised and wild‐caught adults. Overall, these data suggest that (a) hemocyte response to *V. harveyi* is not influenced by the colonization state of the light organ and (b) that an as‐of‐yet unidentified signal, in addition to *V. fischeri* persistence, promotes a fully mature hemocyte response to *V. fischeri.* In *Drosophila*, multiple ligands and signaling pathways have been identified that are required for lymph gland development and hemocyte maturation (Fossett, 2013; Minakhina, Tan, & Steward, 2011; Tan, Goh, & Minakhina, 2012). In addition, injection of β‐1,3‐glucan into the hemocoel of the crayfish *Pacifastacus leniusculus* resulted in hemocyte maturation that occurred in circulation. Although hematopoiesis is yet to be characterized in *E. scolopes*, these findings from other organisms support the hypothesis that hemocyte maturation can be influenced by factors downstream of hematopoiesis through signaling in hemolymph (Söderhäll, Bangyeekhun, Mayo, & Söderhäll, 2003).

### 4.4 Phagocytosis as an early specificity mechanism

We observed that hemocytes isolated from juvenile animals, regardless of symbiotic state, phagocytosed *V. harveyi* yet did not engulf *V. fischeri* in our in vitro assays*.* Previous studies have noted hemocytes in light organ crypt spaces in juvenile animals with internalized bacteria (Casaburi, Goncharenko‐Foster, Duscher, & Foster, 2017; Nyholm & Mcfall‐Ngai, 1998). In one study, phagocytosed *V. fischeri* cells were observed in hemocytes from animals that were maintained under microgravity conditions that likely induced stress responses in the host (Casaburi et al., 2017). Because our study was conducted exclusively in vitro, in vivo conditions within the light organ may differ and also contribute to hemocyte–bacteria interactions. Hemocytes have been shown to migrate into the light organ in both juvenile and adult stages yet whether these hemocytes re‐enter the circulatory system, is currently unknown (Koropatnick et al., 2007; Nyholm & Mcfall‐Ngai, 1998; Schwartzman et al., 2015). Even though our study was conducted exclusively with circulating hemocytes, our data suggest that general juvenile hemocyte binding and phagocytosis behaviors differ during early development in interactions with *V. fischeri* and *V. harveyi*. Further investigation of hemocyte interactions with bacteria under in vivo conditions may reveal mechanisms by which the host's immune system is altered by light organ colonization.

Hemocytes from apo‐raised adults phagocytosed significantly more *V. harveyi* than *V. fischeri*, a response similar to hemocytes from juvenile animals and different from hemocytes from wild‐caught animals. These data suggest that systemic hemocyte maturation continues past 72 hr post‐hatching, as evidenced by the consistency in the number of *V. harveyi* bound in 72‐hr juvenile and adult hosts (Figures 2 and 4), and that differential phagocytosis may play a role in ensuring initial colonization through hemocyte tolerance of the symbiont. The adult immune system of apo‐raised animals, at least in response to *V. fischeri*, may remain in a juvenile‐like state. Because the innate immune system is often characterized by ligand‐PRR binding at the cell surface (Brubaker, Bonham, Zanoni, & Kagan, 2015) and given that *Vibrio* species may have different outer membrane components, these factors may contribute to hemocytes from sym adult animals differentially binding *V. fischeri* and *V. harveyi*. However, our data suggest that the squid's cellular immune system initially uses the process of phagocytosis as a discriminatory mechanism in juveniles and switches to binding of bacteria later in life. There is some previous evidence to suggest phagocytosis is a discriminatory mechanism in innate immune cells. For example, both *Drosophila* S2 cells and mouse Raw 264.7 macrophages phagocytose *Candida albicans* with white cells but not opaque cells, despite the two cell types having identical genomes (Lohse & Johnson, 2008). This switch between cell types in order to avoid host immune response is thought to be a virulence mechanism for *C*. *albicans*, a fungal pathogen. We hypothesize that *E. scolopes* uses different strategies of binding and phagocytosis to interact with *V. fischeri* during development of the squid‐vibrio association. In addition, hemocyte maturation occurs in coordination with other known host developmental processes that promote symbiosis, including but not limited to, hemocyte trafficking to the juvenile light organ (Koropatnick et al., 2004, 2007), remodeling of the light organ (Doino & McFall‐Ngai, 1995; Koropatnick et al., 2004, 2007; Lamarcq & McFall‐Ngai, 1998; Montgomery & McFall‐Ngai, 1994), processing and delivery of chitin to the symbiont for use as a chemotactic substrate and a carbon source (Kremer et al., 2013; Mandel et al., 2012; Schwartzman et al., 2015; Wier et al., 2010), and a transition to diurnal quiescence/nocturnal activity in the host (Koch et al., 2014). Progression through this system of developmental events, triggered by molecular and physical interactions between host and symbiont,leads to a stable and productive association.

## 5 CONCLUSIONS

Taken together, the data presented in this study demonstrate that *E. scolopes* possesses multiple mechanisms to ensure a specific and life‐long interaction with *V. fischeri*. Upon hatching, hemocytes do not appear to phagocytose the symbiont despite binding to *V. fischeri*. This may be a host mechanism to ensure that colonization is successful and persists during the early stages of the association. In the absence of colonization, *V. fischeri* remains resistant to phagocytosis, suggesting that the host's cellular immune response remains primed to allow for eventual colonization. A mature symbiosis in which sustained colonization is maintained, as seen in wild‐caught adult animals, results in an immune response that ensures long‐term maintenance of the symbiosis while allowing for a robust innate immune response against potential nonsymbiotic and/or pathogenic bacteria. Initial hemocyte recognition (or lack thereof) of the symbiont appears to occur at the level of phagocytosis, whereas later in development, recognition appears to be mediated more by binding dynamics. These results, along with previous studies, suggest that the connections between bacterial binding and phagocytosis are complex, and that symbiont tolerance is a result of the sum of these and other innate immune mechanisms. Future research raising both apo and sym juvenile animals to investigate development of the hemocyte response will provide insight into the cues and molecular mechanisms that underlie maturation of the cellular innate immune response to symbiosis.

## CONFLICT OF INTERESTS

None declared.

## AUTHOR CONTRIBUTIONS

BR, SM, and SN conceptualized the study; SN and BR acquired the funding for the study; BR and SM investigated the study; SN supervised the study; BR, SM, and SN wrote and prepared the original draft; BR, SM, and SN reviewed and edited the final manuscript.

## ETHICS STATEMENT

None required.

## Data Availability

Data from this study are available upon reasonable request to the corresponding authors.
